# Efficacy and tolerability of *Guizhi-Shaoyao-Zhimu* decoction in gout patients: a systematic review and Meta-analysis

**DOI:** 10.1080/13880209.2020.1823426

**Published:** 2020-11-18

**Authors:** Qing Zhang, Ruolan Li, Jia Liu, Wei Peng, Wenxiang Fan, Yongxiang Gao, Wei Jin, Chunjie Wu

**Affiliations:** a School of Pharmacy, Chengdu University of Traditional Chinese Medicine, Chengdu, China; b School of Basic Medicine, Chengdu University of Traditional Chinese Medicine, Chengdu, China; c Emergency Department, Hospital of Chengdu University of Traditional Chinese Medicine, Chengdu, China

**Keywords:** Safety evaluation, traditional Chinese medicine, inflammatory arthritis, heterogeneity

## Abstract

**Context:**

*Guizhi-Shaoyao-Zhimu* decoction (GSZD), a famous ancient oriental Chinese prescription, has been widely used for thousands of years to treat ‘arthromyodynia’.

**Objective:**

The clinical studies of GSZD for the treatment of gout were systematically reviewed to evaluate its clinical efficacy and safety.

**Methods:**

All randomized controlled trials (RCTs) related to GSZD and gout were collected starting from the database establishment until 29 February 2020, from the Embase, PubMed, Cochrane Library, Web of Knowledge, VIP and other databases. This systematic review and meta-analysis were performed in strict accordance with the PRISMA (Preferred Reporting Items for Systematic Reviews and Meta-Analysis) statement, and all analysis of the test was completed using Stata (SE12.0) and Revman (5.3).

**Results:**

A total of 535 studies were searched, and 13 studies were included in our meta-analysis (*n* = 1056 participants). Compared with the conventional western medicine treatments, GSZD treatment yielded a significantly increase in the number of clinically effective patients (OR = 3.67, 95%CI = 2.39–5.64, *p* = 0.57), an improved mean reduction in the level of uric acid (MD = −54.06; 95% CI = −69.95 to −38.17). Meanwhile, the levels of erythrocyte sedimentation rate (ESR), C-reactive protein (CRP) and interleukin-6 (IL-6) were also significantly decreased after the GSZD treatment with no increased relative risk of side-effects.

**Conclusions:**

Our present works suggested that GSZD could be considered as an effective alternative remedy for clinical treatment of gout. In addition, it also provides a scientific basis for GSZD to be better applied in clinic in the future.

## Introduction

Gout, a type of chronic inflammatory arthritis, is generally considered to be caused by the monosodium urate (MSU) crystals deposition in joints (Neogi 2016). Currently, epidemiological evidence indicates that gout prevalence of adults is up to approximately 4% in developed countries with a continuous rising tendency, which might be attributable to the shift of diet and lifestyle besides genetic factors (Zeng 2004; Dalbeth et al. 2016; Zhu et al. 2011). It’s known that gout is closely related to the hyperuricaemia caused by the presence of increased urate concentrations in serum. Increasing reports have revealed that gout could induce kidney disorders and destruction of joints. Furthermore, it is also suggested that gout and hyperuricaemia are closely correlated to other serious diseases including hypertension, diabetes mellitus, atherosclerosis, and coronary heart diseases (CHD) (Dalbeth et al. 2016). Currently, non-steroidal anti-inflammatory drugs (NSAIDs) and colchicine are still the most commonly recommended and used agents for clinical management of gout; besides, corticosteroids are also an alternative treatment strategy for curing gout. However, long-term use of these drugs may result in some bothersome adverse effects, including gastrointestinal ulcers, skin rash, osteoporosis, even reproductive toxicities, etc. (Matsui et al. 1997; Burns and Wortmann 2011). Consequently, finding more reliable alternative treatment strategies for gout with low toxicity is still needed.

**Figure 1.**
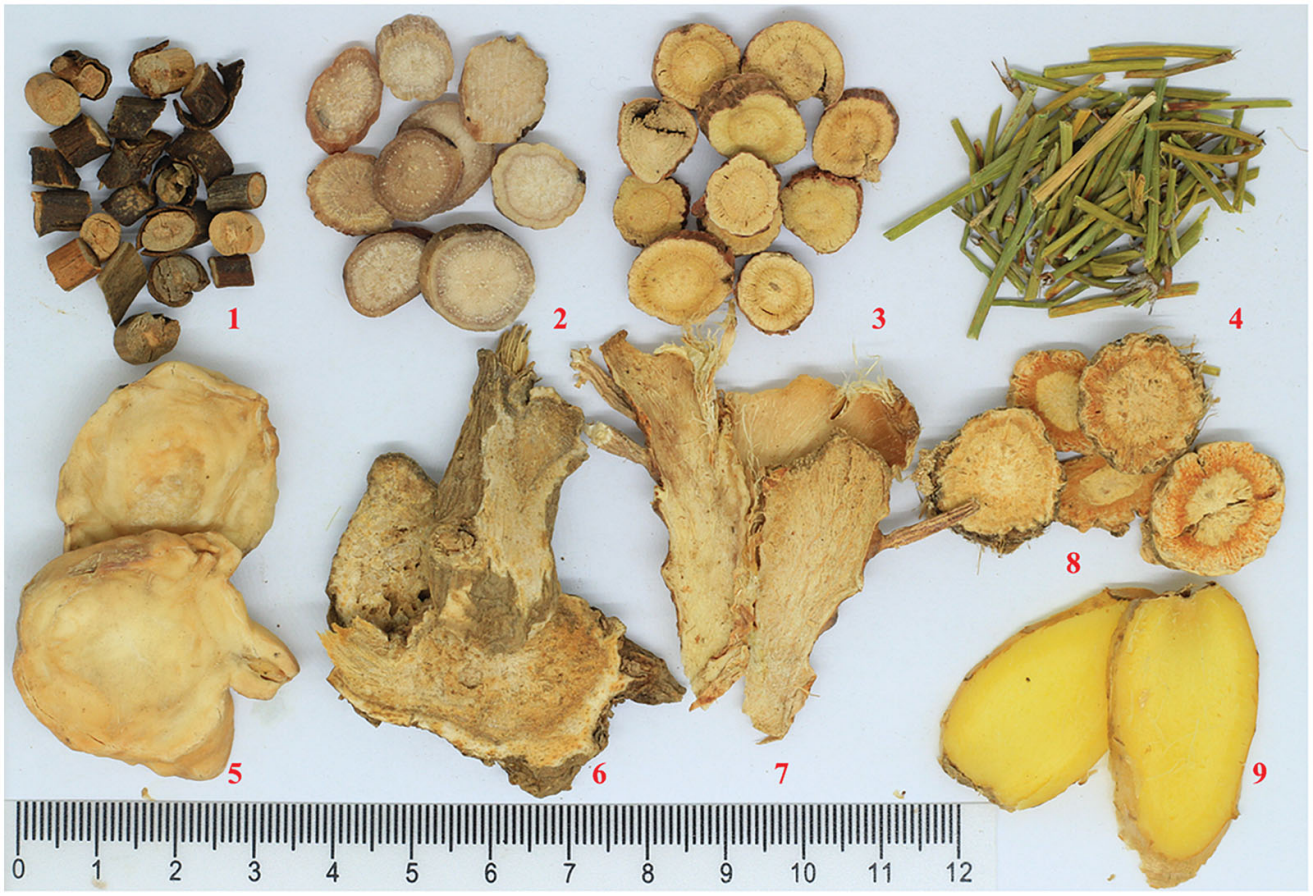
Composition of the *Guizhi-Shaoyao-Zhimu* decoction. 1–9 represent the Cinnamomi Ramulus, Paeoniae Radix Alba, Glycyrrhizae Radix Rhizoma, Ephedrae Herba, Aconm Lateralis Radix Praeparaia, Atractylodes macrocephala Rhizoma, Anemarrhenae Rhizoma, Saposhnikoviae Radix, and Zingiberis Rhizoma Recens, respectively.

*Guizhi-Shaoyao-Zhimu* decoction (GSZD), a famous Chinese prescription first recorded in the *Outline of the Golden Chamber*, is composed of nine herbal medicines, including twigs of *Cinnamomum cassia* (L.) J. Presl (Lauraceae), the radix of *Paeonia lactiflora* Pall. (Paeoniaceae), the rhizome of *Anemarrhena asphodeloides* Bunge (Liliaceae), the radix and rhizome of *Glycyrrhiza uralensis* Fisch. ex DC. (Leguminosae), the whole plant of *Ephedra sinica* Stapf (Ephedraceae), the rhizome of *Zingiber officinale* Roscoe (Zingiberaceae), the rhizome of *Atractylodes macrocephala* Koidz (Asteraceae), the radix of *Saposhnikovia divaricata* (Turcz.) Schischk. (Umbelliferae) and the processed lateralis radix of *Aconitum carmichaeli* Debeaux (Ranunculaceae) (Figure 1). In traditional Chinese medicine (TCM), GSZD is has been widely used in the clinic for treating ‘arthromyodynia’, such as rheumatoid arthritis, gout, osteoarthritis, and other joint diseases (Guo et al. 2016; Daily et al. 2017) for thousands of years. Nowadays, accumulating clinical evidence has indicated GSZD can significantly ameliorate the clinical symptoms of gout and can significantly improve pathological changes in the joints of gout patients; thus, GSZD is considered as an important alternative choice for clinical treatment of gout in TCM (Shi 2012; Wen 2012; Qu et al. 2015; Xie and Chen 2018). Furthermore, animal experimental results also demonstrated that GSZD can significantly alleviate the pathological changes of synovial tissues in sodium urate induced gout rats by decrease of inflammatory cytokines though down-regulating Toll-MyD88 and NF-κB (Li et al. 2013; Fang et al. 2016; Wang et al. 2016).

Currently, increasing research has revealed that some alternative and complementary traditional medicines, such as TCMs, are a feasible approach for curing some difficult miscellaneous diseases. Although, GSZD has been extensively used in clinics for treating gout, most of the research regarding GSZD are anecdotal and have not been systemically investigated with rigorous scientific trials. Consequently, a systematic review and meta-analysis was designed to systemically and objectively evaluate the curative effects and safety of GSZD for gout treatment, which would be beneficial for the reasonable application of this known TCM classic formula for treating gout in clinics.

## Methods

This systematic review and meta-analysis were performed in strict accordance with the PRISMA (Preferred Reporting Items for Systematic Reviews and Meta-Analysis) statement, and all analysis of the test was completed using the software of Stata (SE12.0) and Revman (5.3).

### Search strategy and selection criteria

All randomized controlled trials (RCTs) related to GSZD and gout were collected from the database establishment until 29 February 2020, with no languages restrictions. In our present study, the following document libraries were used: Embase, PubMed, Cochrane Library, Web of Knowledge, VIP, Chinese Biomedical Database (CBM), Wanfang and China National Knowledge Infrastructure (CNKI), journals and dissertations are included. The following key terms were used to search the relevant studies: *Guizhi-Shaoyao-Zhimu* (*Guizhi-Shaoyao-Zhimu* in Chinese Pinyin), *Guizhi-Shaoyao-Zhimu* decoction (*Guizhi-Shaoyao-Zhimu* Tang in Chinese Pinyin), GSZD, gout (Tong Feng or Li Jie Feng or Tong Feng Shi in Chinese Pinyin), hyperuricaemia (Gao Niao Suan Xue Zheng in Chinese Pinyin) and randomized controlled trial (Lin Chuang Sui Ji Dui Zhao Shi Yan in Chinese Pinyin). Literature retrieval was performed in the following combinations: (*Guizhi-Shaoyao-Zhimu* decoction or Gui Shao Zhimu decoction or GSZD or *Cassia Twig* or *Paeonia lactiflora* or *Anemarrhena Rhizome* or *Ramulus Cinnamomi cassia* Presl) and (gout or hyperuricaemia or gouts) and (randomized controlled trial or RCT or Clinical trial) for English databases. (*Guizhi-Shaoyao-Zhimu* or *Guizhi-Shaoyao-Zhimu* Tang or Gui Shao Zhimu or GSZD) and (Tong Feng or Gao Niao Suan Xue Zheng or Li Jie Feng or Tong Feng Shi) and (Lin Chuang Sui Ji Dui Zhao Shi Yan or RCT) for Chinese databases. Meanwhile, we also manually searched the list of references for eligible articles. In addition, grey literature was searched to ensure that the search was complete enough to avoid publication bias. Literature retrieval was performed by only two researchers.

### Inclusion criteria

Eligible literature must meet the following criteria: (1) participants included in the randomized controlled trial are adults, excluding children and pregnant women, and must meet internationally recognized diagnostic criteria for gout; (2) GSZD is used alone or in combination with conventional treatment to compare with conventional treatment; (3) outcome measurement indicators include the number of patients reaching clinical efficacy (NPE), uric acid (UA), erythrocyte sedimentation rate (ESR), C-reactive protein (CRP), interleukin-6 (IL-6) and one or more adverse reactions; (4) There were no other therapeutic factors between the treatment group and the control group.

### Exclusion criteria

Exclusion criteria are as follows: (1) duplicate publications, data errors, incomplete or unavailable data; (2) *in vitro* and *in vivo* experiments on non-human species; (3) non-randomized controlled trials, observation and retrospective studies, case reports, expert experience and comments; (4) the secondary research with gout as an experiment and gout concurrent with other costs.

Literature screening was performed independently by two researchers (the source, author, funding project and other information were hidden). According to the screening criteria described above, the title and abstract of the study were first observed, and excluded the apparently unrelated literature. Then we further read the full text in depth to confirm whether it was qualified literature or not. The retrieved literature and the literature number were cross-checked. If there was any inconsistency, it was determined by a third party.

### Data extraction

Experimental data met the inclusion criteria as follows: baseline information in the study, including the first author, publication time, total number of patients, age, sex, duration of illness, details and duration of intervention, adverse events, NPE, mean values and standard deviation (SD) of UA, ESR, CRP, IL-6 at the end of intervention.

The clinical efficacy between GSZD and conventional western medicine for the gout treatment was compared. In our present paper, the changes of uric acid, ESR, CRP and IL-6 were considered as the main clinical results, whilst the total number of clinically effective patients was considered as the secondary results, and adverse events were considered as the main indicators of safety evaluation.

### Risk of bias in individual studies

We evaluated the risk assessment method of bias according to the method of Cochrane collaboration network, including Random sequence generation (i.e., selection bias); Allocation concealment (i.e., selection bias); blinding of participants and personnel (i.e., performance bias); Blinding of the outcome assessment (i.e., detection bias); incomplete outcome data (i.e., attrition bias); Selective reporting (i.e., reporting bias) and other bias (i.e., other bias).

Based on the actual situation of the literature, the two researchers assessed the risk of bias in the included literature (hidden journal source, author, fund project and other information). The above seven results are, respectively, used as ‘yes’, ‘no’ and ‘uncertainty’ to represent the low deviation risk, high deviation risk and uncertain deviation risk. If the evaluation results are inconsistent, it shall be discussed by two people to solve the problem; otherwise, it shall be discussed by a third party to reach an agreement. Complete deviation risk diagram with Revman (5.3).

### Publication of risk analysis of bias

For result in the meta-analysis (the number of studies is no fewer than 10), Egger test and Begg test were used to detect the publication bias of the included test, and *p* value less than 0.1 was defined as a significant publication bias. Meanwhile, we also established funnel plots and observed their symmetry to evaluate whether there were publication deviations.

### Sensitivity analysis

We took the NPE and adverse events as dichotomous variables and calculated their Odds Ratio (OR) or Relative Risk (RR). UA, ESR, CRP and IL-6 indexes were taken as continuous variables, and standardized mean value (SMD) and mean difference (MD) were used as effect indexes. All data were weighted and combined, and the point estimates and 95% confidence intervals (CI) of the combined effects were given. At the same time, we used subgroup analysis to exclude significant clinical heterogeneity according to different intervention strategies. Chi-square test was used to determine whether statistical heterogeneity existed between studies. *p* < 0.1 was used as the criterion for heterogeneity. We also conducted an *I*^2^ test to assess the magnitude of statistical heterogeneity between studies, with values greater than 50% considered indicators of moderate to high heterogeneity. In the absence of statistical heterogeneity, fixed-effect models were used to estimate total RR, WMD (or SMD) and 95% CI. Otherwise, the random-effect model is adopted, and the results are not combined, and only descriptive analysis is conducted. For each result in the meta-analysis, we used sensitivity analysis to assess the stability of the combined results. Exclude the included studies one by one, and reanalyze the remaining studies. Compare the difference between the new merge and the exclude merge, and we used Stata (SE12.0) and Revman (5.3) for all statistical analysis.

## Results

### Study characteristics

**Figure 2.**
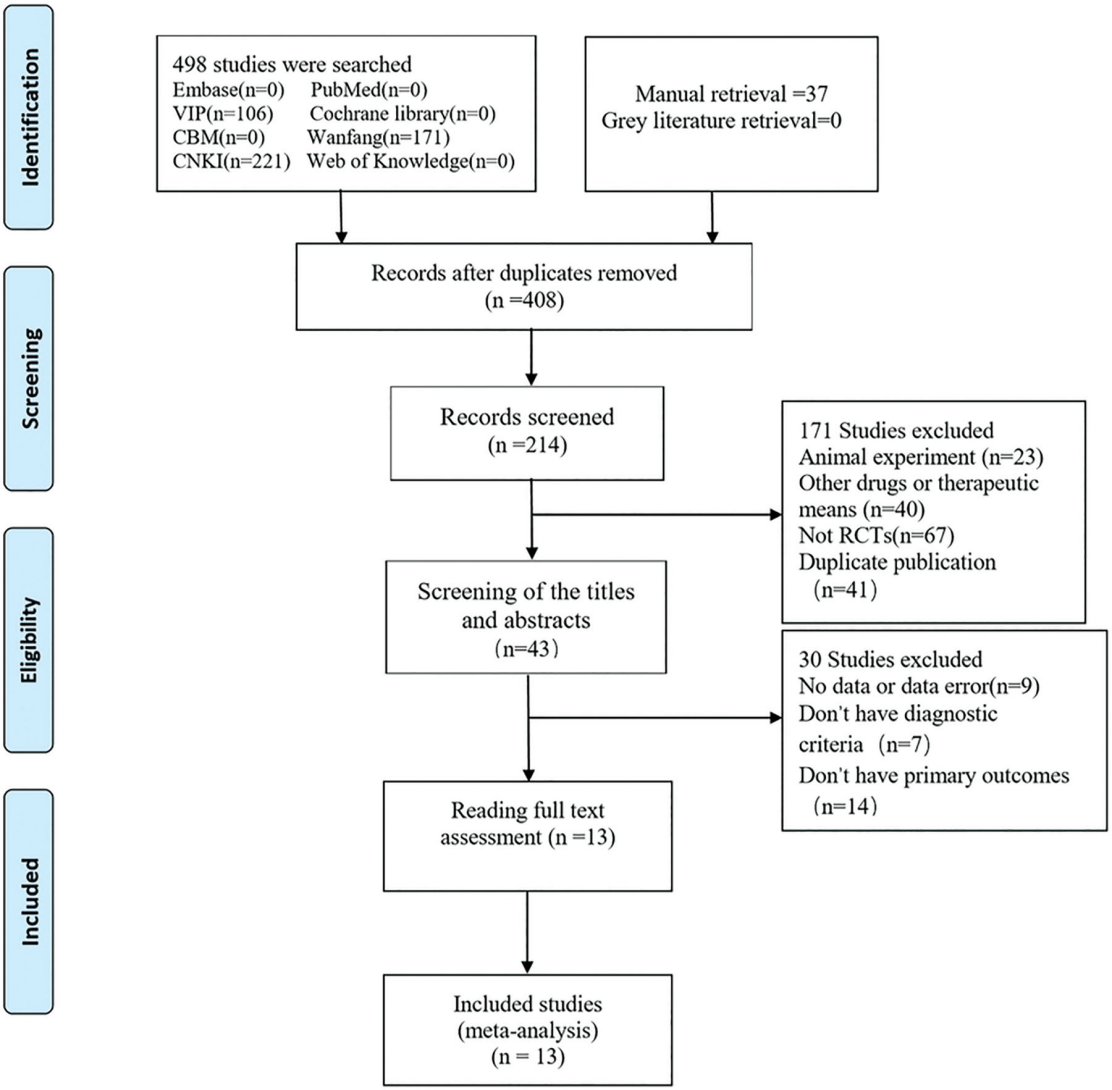
Flow chart of study selection.

In the initial search, a total of 535 articles were collected in our study, excluding 127 duplicate, 408 remained. After screening, 194 were discarded, and subsequently 171 were further discarded after a preliminary screening of the titles and abstracts. After reading the full text, 30 articles were further excluded, and finally 13 publications (*n* = 1056 participants) were enrolled in our analysis, and no grey literature was retrieved. The literature screening process is shown in Figure 2. All 13 studies (Xu and Chen 1998; He et al. 2013; Hu and Luo 2013; Shen et al. 2014; He 2015; Wang 2015; Luo 2017; Yu et al. 2017; Chen 2018; Li et al. 2018; Xie and Chen 2018; Qiao 2019; Zhu 2019) were journal articles and conducted in China and published from 1998 to 2020. Amongst these 13 studies, only one study (Li et al. 2018) had patients falling off and discontinued, and all the other 12 studies were completed. In the present study, seven RCTs were GSZD treatment alone (Xu and Chen 1998; He et al. 2013; Shen et al. 2014; He 2015; Luo 2017; Li et al. 2018; Xie and Chen 2018), and other six RCTs were combined treatment by GSZD and conventional western medicine (CWM) (Hu and Luo 2013; Wang 2015; Yu et al. 2017; Chen 2018; Qiao 2019; Zhu 2019). Three studies did not explicitly report patient diagnostic criteria for gout (Wang 2015; Luo 2017; Xie and Chen 2018), and the remaining studies used scientific diagnostic criteria, such as the diagnostic criteria of American College of Rheumatology (ACR) in 1997 and the diagnostic criteria of European League Against Rheumatism (EULAR) in 2011. The intervention times were ranging from 1 week to 24 weeks with the average time of 4.8 weeks, and the baseline characteristics and details of the included studies are shown in Tables 1–3.

**Table 1.** The characteristics of the included RCTs.

	Cases		Sex male/female	Age(years) mean	Disease course, range, mean	Diagnostic criteria	Reference
No	T	C					
1	30	30	24/36	52.8	2.1y	ACR 1997	Yu et al. (2017)
2	40	40	T:35/5 C:36/4	T:45.1 C:44.8	T:2.8y C:2.7 y	NA	Wang (2015)
3	30	30	T:28/2 C:29/1	T:44.1 C:41.8	T:2.9y C:3.2y	ACR 1997	He et al. (2013)
4	35	35	T:29/6 C:30/5	T:46.5 C:47.2	T:2d-20y C:3d-21y	ACR 1997	Shen et al. (2014)
5	45	45	T:34/11 C:31/14	T:46.36 C:45.78	T:3d-12y C:7d-16y	ACR 1997	He (2015)
6	34	34	T:16/18 C:20/14	T:54.5 C:56.1	T:2.1y C:2.5y	NA	Luo (2017)
7	74	74	T:59/15 C:57/17	T:56.7 C:55.5	T:11.5y C:11.5y	ACR 1997	Li et al. (2018)
8	30	30	T:19/11 C:17/13	T:44.2 C:43.5	T:0.67y-3y C:1y-3y	NA	Xie and Chen (2018)
9	46	46	T:29/17 C:30/16	T:43.9 C:42.6	T:43.6m C:47.9m	EULAR 2011	Chen (2018)
10	40	40	T:32/8 C:34/6	T:44.5 C:44.8	T:47.8m C:48.4m	ACR 1997	Hu and Luo (2013)
11	45	38	T:44/1 C:36/2	T:46.3 C:47.8	T:1d-22y C:2d-18y	ACR 1997	Xu and Chen (1998)
12	45	45	T:39/6 C:40/5	T:45.65 C:45.7	NA	ACR 1997	Zhu (2019)
13	43	43	T:39/4 C:41/2	T:40.07 C:39.22	T:4.5y C:4.78y	ACR 1997	Qiao (2019)

T: trail group; C: control group; d: day; m: month; y: year; ACR: American college of rheumatology; EULAR: European league against rheumatism; NA: not available.

**Table 2.** Intervention and adverse events of included studies.

	Study design	Intervention		Duration (week)	Adverse events	Outcome measures	
		T	C				
1	RCT	GSZD + CWM	Colchicine	4		NPE, UA, ESR, CRP	Yu et al. (2017)
2	RCT	GSZD + CWM	Celecoxib capsules	4		NPE, UA, ESR, CRP	Wang (2015)
3	RCT	GSZD	Celecoxib capsules	4		NPE, UA, ESR, CRP	He et al. (2013)
4	RCT	GSZD	Colchicine	1	T:0/C:32	NPE, UA, ESR, IL-6	Shen et al. (2014)
5	RCT	GSZD	Colchicine	2		NPE, UA, ESR, CRP	He (2015)
6	RCT	GSZD	Celecoxib capsules	4		NPE, UA, ESR, CRP	Luo (2017)
7	RCT	GSZD	Allopurinol	24	T:3/C:17	NPE, UA	Li et al. (2018)
8	RCT	GSZD	Diclofenac	2		NPE, UA, ESR, CRP	Xie and Chen (2018)
9	RCT	GSZD + CWM	Febuxostat Tablets	12		UA, IL-6	Chen (2018)
10	RCT	GSZD + CWM	Colchicine	2		NPE, IL-6	Hu and Luo (2013)
11	RCT	GSZD	Colchicine	1		NPE, UA	Xu and Chen (1998)
12	RCT	GSZD + CWM	Celecoxib + Colchicine	1	T:4/C:11	NPE, CRP, IL-6, ESR	Zhu (2019)
13	RCT	GSZD + CWM	Etoncoxib + Sodium bicarbonate	2	T:1/C:1	NPE, UA, ESR, CRP	Qiao (2019)

T: trail group; C: control group; RCT: randomized controlled trial; GSZD: Guizhi-Shaoyao-Zhimu decocation; CWM: conventional western medicine; NPE: the number of patients reaching clinical efficacy; UA: uric acid; ESR: erythrocyte sedimentation rate; CRP: C-reactive protein; IL-6: interleukin 6.

**Table 3.** Detail information of included studies.

	Compositions	Preparation	Administration	Reference
1	Twigs of *Cinnamomum cassia* (20 g), Radix of *Cynanchum otophyllum* (15 g), Rhizoma of *Anemarrhena asphodeloides* (15 g), Radix and Rhizoma of *Glycyrrhiza uralensis* (6 g), Whole plant of *Ephedra sinica* (15 g), Rhizoma of *Zingiber officinale* (5 g), Rhizoma of *Atractylodes macrocephala* (12 g), Radix of *Saposhnikovia divaricate* (6 g), Cortex of *Phellodendron chinense* (20 g), Seeds of *Coix lacryma-jobi* (12 g),Whole plant of *Lysimachia christinae* (10 g), Cortex of *Paeonia suffruticosa* (10 g）	Decoction with water	Oral, twice a day	Yu et al. (2017)
2	Twigs of *Cinnamomum cassia* (15 g), Radix of *Cynanchum otophyllum* (15 g), Rhizoma of *Anemarrhena asphodeloides* (15 g), Whole plant of *Ephedra sinica* (9 g), Rhizoma of *Atractylodes macrocephala* (15 g), Radix of *Saposhnikovia divaricate* (12 g), Processed lateralis Radix of *Aconitum carmichaeli* (10 g), Rhizoma of *Asarum sieboldii* (6 g), Seeds of *Coix lacryma-jobi* (30 g), Rhizoma of *Alisma orientale* (15 g), Rhizoma of *Dioscorea hypoglauca* (15 g), Rhizoma of *Smilax glabra* (15 g), Radix of *Cyathula officinalis* (15 g), Radix of *Angelica pubescens* (12 g)	Decoction with water	Oral, twice a day	Wang (2015)
3	Twigs of *Cynanchum cassia* (15 g), Radix of *Cynanchum otophyllum* (15 g), Rhizoma of *Anemarrhena asphodeloides* (15 g), Processed lateralis Radix of *Aconitum carmichaeli* (10 g), Rhizoma of *Atractylodes macrocephala* (15 g), Whole plant of *Ephedra sinica* (9 g), Radix of *Saposhnikovia divaricate* (12 g), Rhizoma of *Asarum sieboldii* (6 g), Seeds of *Coix lacryma-jobi* (30 g), Radix of *Cyathula officinalis*, Radix of *Angelica pubescens* (12 g), Rhizoma of *Smilax glabra* (15 g), Rhizoma of *Dioscorea hypoglauca* (15 g), Rhizoma of *Alisma orientale* (15 g)	Decoction with water	Oral, twice a day	He et al. (2013)
4	Twigs of *Cynanchum cassia* (20 g), Radix of *Cynanchum otophyllum* (15 g), Rhizoma of *Anemarrhena asphodeloides* (20 g), Radix and Rhizoma of *Glycyrrhiza uralensis* (10 g), Rhizoma of *Zingiber officinale* (25 g), Whole plant of *Ephedra sinica* (10 g)、Rhizoma of *Atractylodes macrocephala* (25 g), Radix of *Saposhnikovia divaricate* (20 g), Processed lateralis Radix of *Aconitum carmichaeli* (20 g), Twigs of *Morus alba* (10 g), Radix of *Achyranthes bidentata* (30 g), Rhizoma of *Smilax glabra* (30 g), Rhizoma of *Dioscorea hypoglauca* (30 g)	Decoction with water	Oral, twice a day	Shen et al. (2014)
5	Twigs of *Cynanchum cassia* (9 g), Radix of *Cynanchum otophyllum* (15 g), Rhizoma of *Anemarrhena asphodeloides* (12 g), Whole plant of *Ephedra sinica* (6 g), Radix and Rhizoma of *Glycyrrhiza uralensis* (6 g), Rhizoma of *Zingiber officinale* (6 g), Rhizoma of *Atractylodes macrocephala* (9 g), Radix of *Saposhnikovia divaricate* (9 g), Processed lateralis Radix of *Aconitum carmichaeli* (9 g), Seeds of *Coix lacryma-jobi* (30 g), Radix and Rhizoma of *Glycyrrhiza uralensis* (6 g)	Decoction with water	Not provided	He (2015)
6	Twigs of *Cynanchum cassia* (15 g), Radix of *Cynanchum otophyllum* (15 g), Rhizoma of *Anemarrhena asphodeloides* (18 g), Radix and Rhizoma of *Glycyrrhiza uralensis* (10 g), Radix of *Saposhnikovia divaricate* (12 g), Rhizoma of *Zingiber officinale* (15 g), Rhizoma of *Atractylodes macrocephala* (15 g) , Processed lateralis Radix of *Aconitum carmichaeli* (8 g), Folium of *Perilla frutescens* (15 g), *Poria cocos* (15 g)	Decoction with water	Oral, twice a day	Luo (2017)
7	Twigs of *Cynanchum cassia* (10 g), Radix of *Cynanchum otophyllum* (20 g), Rhizoma of *Anemarrhena asphodeloides* (10 g), Radix and Rhizoma of *Glycyrrhiza uralensis* (6 g), Rhizoma of *Atractylodes macrocephala* (15 g), Processed lateralis Radix of *Aconitum carmichaeli* (10 g), Radix of *Codonopsis pilosula* (20 g), Rhizoma of *Atractylodes lancea* (15 g), Cortex of *Magnolia officinalis* (10 g), Pericarpium of *Citrus reticulate* (15 g), Seeds of *Coix lacryma-jobi* (30 g), Rhizoma of *Dioscorea hypoglauca* (15 g), Rhizoma of *Curcuma longa* (10 g), Radix of *Cyathula officinalis* (15 g), Caulis of *Spatholobus suberectus*（20 g）, Rhizoma of *Alisma orientale* (15 g)	Decoction with water	Oral, twice a day	Li et al. (2018)
8	Twigs of *Cynanchum cassia* (15 g), Radix of *Cynanchum otophyllum* (15 g), Rhizoma of *Anemarrhena asphodeloides* (15 g), Radix of *Saposhnikovia divaricate* (12 g), Rhizoma of *Atractylodes macrocephala* (15 g), Processed lateralis Radix of *Aconitum carmichaeli* (10 g), Whole plant of *Ephedra sinica* (9 g), Rhizoma of *Asarum sieboldii* (6 g), Seeds of *Coix lacryma-jobi* (30 g), Rhizoma of *Alisma orientale* (15 g), Rhizoma of *Smilax glabra* (15 g), Rhizoma of *Dioscorea hypoglauca* (15 g), Radix of *Cyathula officinalis* (15 g), Radix of *Angelica pubescens* (12 g)	Decoction with water	Oral, twice a day	Xie and Chen (2018)
9	Twigs of *Cynanchum cassia* (12 g), Radix of *Cynanchum otophyllum* (9 g), Rhizoma of *Anemarrhena asphodeloides* (12 g), Rhizoma of *Atractylodes macrocephala* (12 g), Radix of *Saposhnikovia divaricate* (8 g), Rhizoma of *Zingiber officinale* (8 g), Whole plant of *Ephedra sinica* (6 g ), Processed lateralis Radix of *Aconitum carmichaeli* (25 g), Radix and Rhizoma of *Glycyrrhiza uralensis* (6 g)	Decoction with water	Oral, twice a day	Chen (2018)
10	Twigs of *Cynanchum cassia* (12 g), Radix of *Cynanchum otophyllum* (9 g), Rhizoma of *Anemarrhena asphodeloides* (12 g), Radix of *Saposhnikovia divaricate* (8 g), Whole plant of *Ephedra sinica* (6 g), Rhizoma of *Atractylodes macrocephala* (12 g), Rhizoma of *Zingiber officinale* (8 g), Radix and Rhizoma of *Glycyrrhiza uralensis* (6 g), Processed lateralis Radix of *Aconitum carmichaeli* (25 g)	Decoction with water	Oral, twice a day	Hu and Luo (2013)
11	Twigs of *Cynanchum cassia* (10 g), Radix of *Cynanchum otophyllum* (30 g), Rhizoma of *Anemarrhena asphodeloides* (10 g), Whole plant of *Ephedra sinica* (10 g), Processed lateralis Radix of *Aconitum carmichaeli* (10 g), Rhizoma of *Atractylodes macrocephala* (20 g), Radix and Rhizoma of *Glycyrrhiza uralensis* (6 g), Rhizoma of *Zingiber officinale* (6 g), Radix of *Stephania tetrandra* (10 g)	Decoction with water	Not provided	Xu and Chen (1998)
12	Twigs of *Cynanchum cassia* (20 g), Rhizoma of *Anemarrhena asphodeloides* (20 g), Whole plant of *Ephedra sinica* (10 g), Processed lateralis Radix of *Aconitum carmichaeli* (20 g), Rhizoma of *Atractylodes macrocephala* (25 g), Radix and Rhizoma of *Glycyrrhiza uralensis* (10 g), Rhizoma of *Smilax glabra* (30 g), Rhizoma of *Dioscorea hypoglauca* (30 g), Radix of *Saposhnikovia divaricate* (20 g), Radix of *Cyathula officinalis* (30 g), Rhizoma of *Zingiber officinale* (25 g)	Decoction with water	Oral, twice a day	Zhu (2019)
13	Twigs of *Cinnamomum cassia* (12 g), Rhizoma of *Anemarrhena asphodeloides* (12 g), Radix and Rhizoma of *Glycyrrhiza uralensis* (6 g), Whole plant of *Ephedra sinica* (6 g), Rhizoma of *Zingiber officinale* (15 g), Processed lateralis Radix of *Aconitum carmichaeli* (10 g), Radix of *Cyathula officinalis* (15 g), Seeds of *Coix lacryma-jobi* (30 g)	Decoction with water	Oral, three times a day	Qiao (2019)

GSZD: Guizhi-shaoyao-zhimu decocation.

### Risk of bias

**Figure 3.**
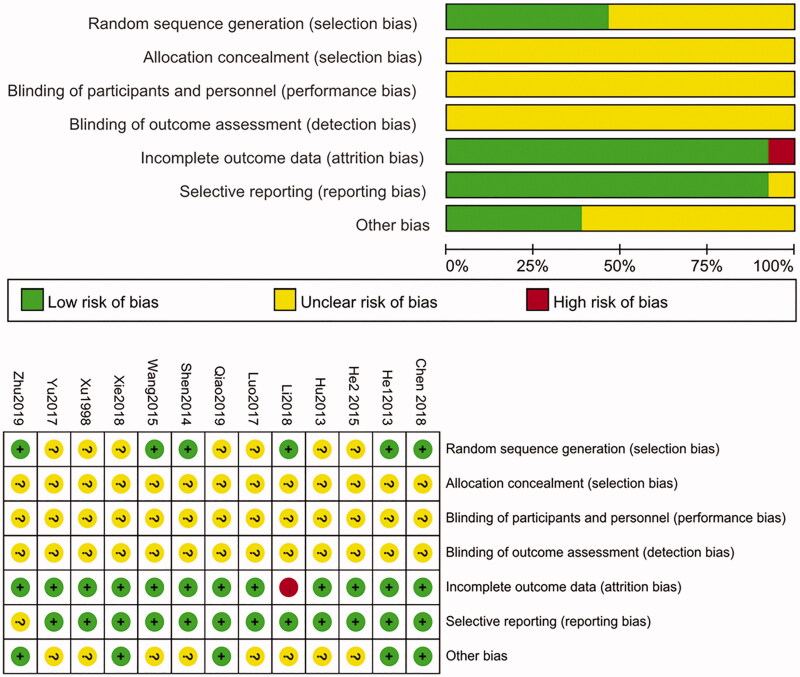
Risk of bias graph in all 13 RCTs included in the systematic.

The risk deviations of the included studies were assessed in the present paper. Overall, most of the trials included have low to medium qualities, and 6 trials (He et al. 2013; Shen et al. 2014; Wang 2015; Chen 2018; Li et al. 2018; Zhu 2019) used the random allocation with random number table. But none of the studies described the randomization hiding method, and none of the experiments described the correlation blind method. Thus, risks of bias of all the collected papers were decided as uncertain deviation risk. This may be due to the particularity and characteristic of individualized treatment based on syndrome differentiation in TCM remedy, and it is difficult to achieve completely blind design in clinical practice. The researchers divided the patients into the treatment group and control group, and finally evaluated the effect of intervention by comparing the objective outcome indicators between the two groups, which can appropriately reduce the deviation caused by blind method. There was a study (Li et al. 2018) in which patients fell off and so on; no cases were selectively reported and the outcome data were complete, and other biases are unclear. The risk profile for each study is shown in Figure 3.

### Outcome measures

#### The secondary indicator of NPE

**Figure 4.**
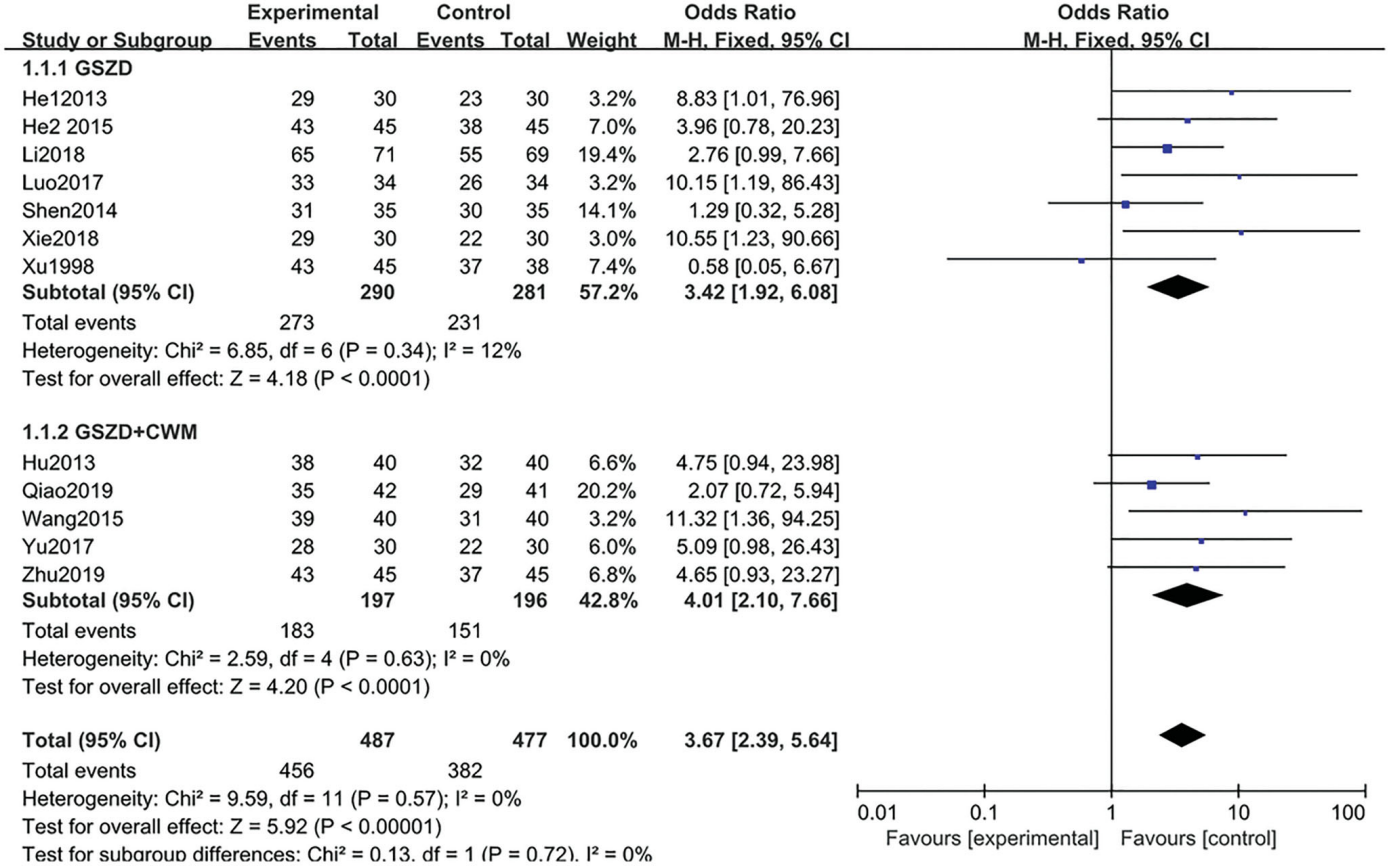
Forest plot of the number of patients reaching clinical efficacy.

A total of 12 studies (Xu and Chen 1998; He et al. 2013; Hu and Luo 2013; Shen et al. 2014; He 2015; Wang 2015; Luo 2017; Yu et al. 2017; Li et al. 2018; Xie and Chen 2018; Qiao 2019; Zhu 2019) (*n* = 964) reported the main outcome index of NPE. Among these reports, seven included a trial group with GSZD treatment alone, and the remaining five RCTs reported a trial group with combination treatment of GSZD and CWMs. The fixed-effect model (*p* = 0.57) was used to summarize and analyze the data from the 12 trials. Compared with the CWM group, the proportion of subjects who received NPE at the final visit was higher (456/487, 93.63% vs. 382/477, 80.08%) in the GSZD-treatment group (OR = 3.67, 95% CI = 2.39–5.64, *p* = 0.57), and there is no heterogeneity between these studies (Figure 4).

**Figure 5.**
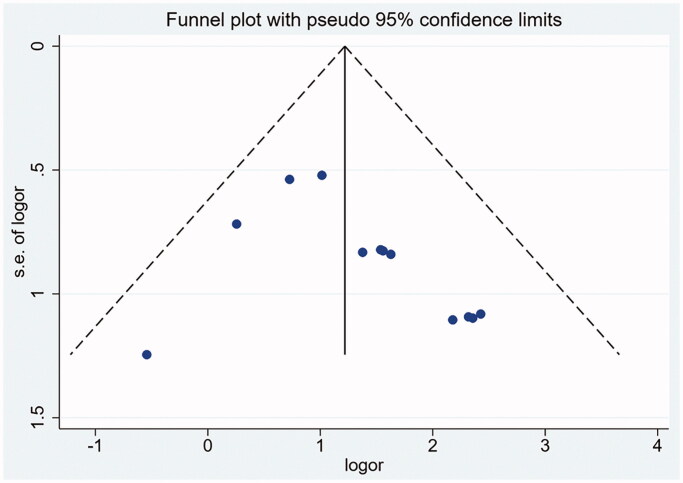
Funnel plot of the number of patients reaching clinical efficacy.

The relatively symmetrical funnel plot showed that there was no publication bias (Figure 5), and further analysis of Begg test and Egger test also found that there was no publication bias (Table 4). The included studies were deleted one by one for sensitivity analysis, and the remaining studies were used to compare the new merged effect with the original one without obvious difference, showing that the merged results were highly stable.

**Table 4.** The results of statistical analysis.

Outcome measures	Analytical model	Effect size	Subgroup analysis	Point estimates	95% confidence intervals (CI)	Heterogeneity		Publication bias	
						*I* ^2^	*p*	Begg	Egger
NPE	Fixed-effect model	OR	GSZD	3.42	1.92, 6.08	12%	0.34	0.115	0.112
			GSZD + CWM	4.01	2.10, 7.66	0%	0.63		
			Total	3.67	2.39, 5.64	0%	0.57		
UA	Random-effect model	MD	GSZD	−64.90	−83.83, −45.45	57%	0.03	/	0
			GSZD + CWM	−36.28	−52.58, −19.98	70%	0.02		
			Total	−54.06	−69.95, −38.17	84%	0.000		
ESR	Random-effect model	SMD	GSZD	−0.30	−0.56, −0.03	35%	0.19		
			GSZD + CWM	−0.78	−1.35, −0.21	83%	0.0006		
			Total	−0.52	−0.84, −0.20	76%	0.298		
CRP	Fixed-effect model	WMD	GSZD	−1.63	−2.36, −0.97	38.00%	0.17		
			GSZD + CWM	−1.63	−1.88, −1.37	0%	0.57		
			Total	−1.63	−1.87, −1.39	6%	0.38		
IL-6	Random-effect model	SMD	GSZD	−0.40	−0.88, 0.07	0	0.10		
			GSZD + CWM	−0.83	−1.05, −0.41	48%	0.15		
			Total	−0.73	−1.05, −0.41	52%	0.10		
Safety evaluation	Random-effect model	RR	GSZD	0.15	0.03, 0.68	69%	0.04		

GSZD: Guizhi-shaoyao-zhimu decocation; CWM: conventional western medicine; RR: risk ratio; WMD: weighted mean difference; SMD: standardized mean difference; NPE: the number of patients reaching clinical efficacy; UA: uric acid; ESR: erythrocyte sedimentation rate; CRP: C-reactive protein; IL-6: interleukin 6.

#### The main indicators of UA, ESR, CRP and IL-6

**Figure 6.**
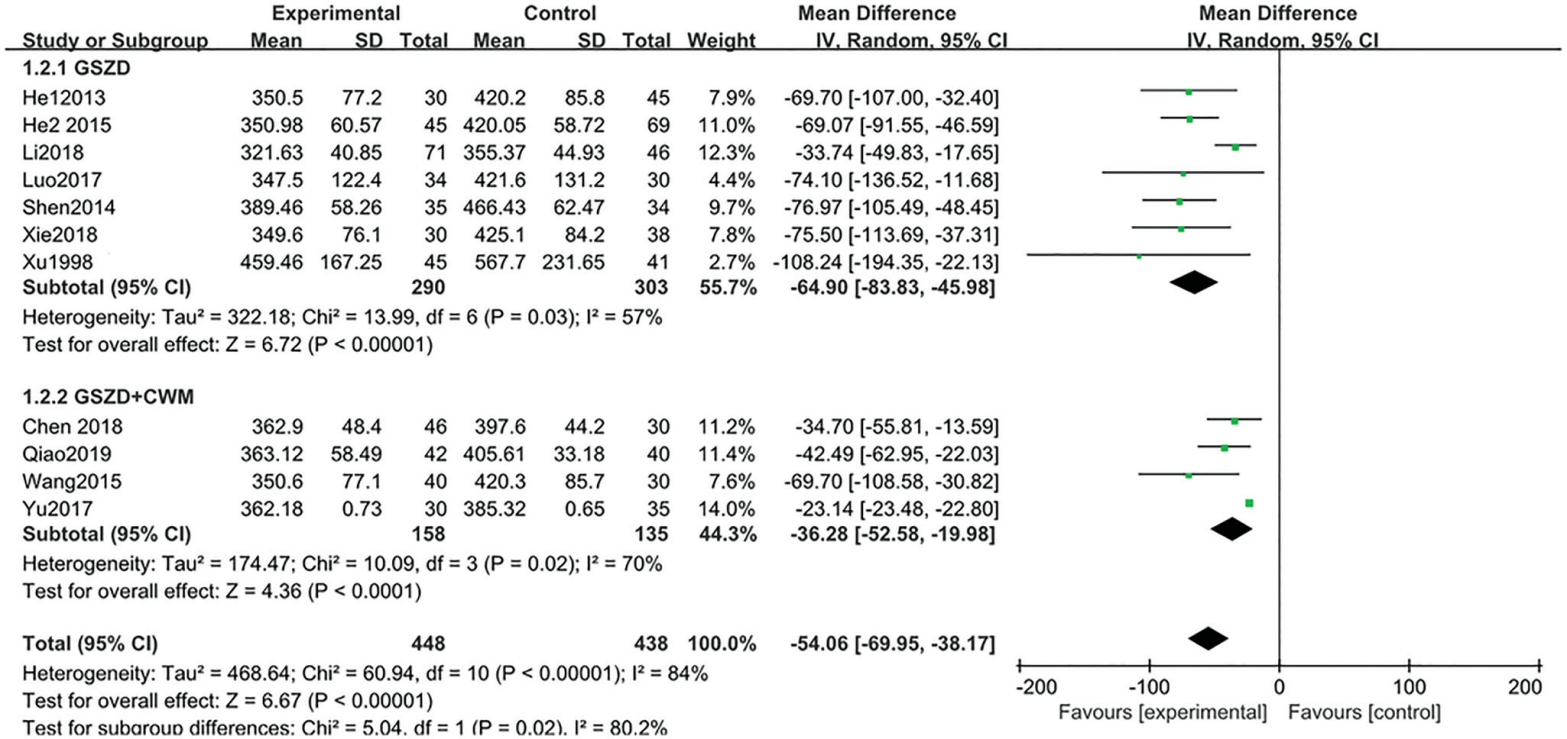
Forest plot of the uric acid.

Eleven studies (Xu and Chen 1998; He et al. 2013; Shen et al. 2014; He 2015; Wang 2015; Luo 2017; Yu et al. 2017; Chen 2018; Li et al. 2018; Xie and Chen 2018; Qiao 2019) (*n* = 886) were summarized and analyzed, and these studies reported changes in UA at the end of the intervention. From the combined results, compared with the CWM group, the UA of patients in the GSZD group significantly decreased (MD = −64.90, CI = −83.83 to −45.98, *p* = 0.03) at the end of intervention, similarly, the UA levels in the GSZD + CWM group also significantly decreased (MD = −36.28, CI = −52.58 to −19.98, *p* = 0.02), with statistically significant heterogeneity amongst these studies (Figure 6), and this may be related to the differences in patients' physical signs, drug dosage and intervention time. Therefore, the random-effect model was used for combined analysis. Furthermore, the result of Begg test and Egger test showed that there were obvious publication biases in this analysis (Table 4). No significant difference before and after the exclusion study was observed in the sensitivity analysis.

**Figure 7.**
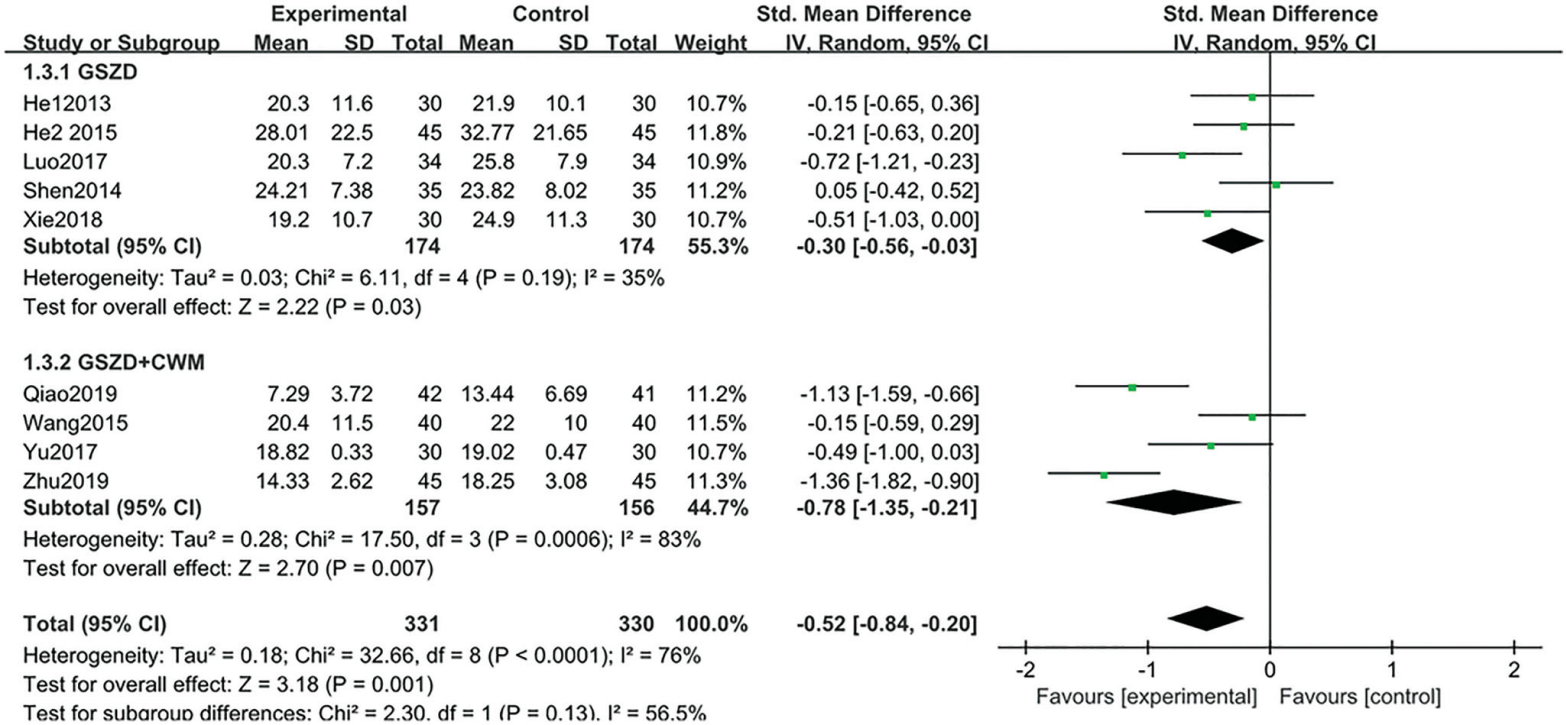
Forest plot of erythrocyte sedimentation rate.

Nine included studies (He et al. 2013; Shen et al. 2014; He 2015; Wang 2015; Luo 2017; Yu et al. 2017; Xie and Chen 2018; Qiao 2019; Zhu 2019) (*n* = 661) were designed to measure changes in ESR at the end of treatment. After combined analysis of these data, we found that GSZD group can significantly reduce the ESR, compared to CWM group (SMD = −0.30, CI = −0.56 to −0.03, *p* = 0.19), and there was no statistical heterogeneity between these studies. Similarly, when GSZD was combined with CWM, ESR levels were significantly reduced (SMD = −0.78, CI = −1.35 to −0.21, *p* = 0.0006). Despite statistical heterogeneity between these studies which might be related to differences in the drug dosage, duration of the study, and the underlying conditions of the participants, and the results of each study suggested that the addition of GSZD therapy is beneficial for ESR management (Figure 7). In addition, the results of sensitivity analysis showed the combined results were highly stable.

**Figure 8.**
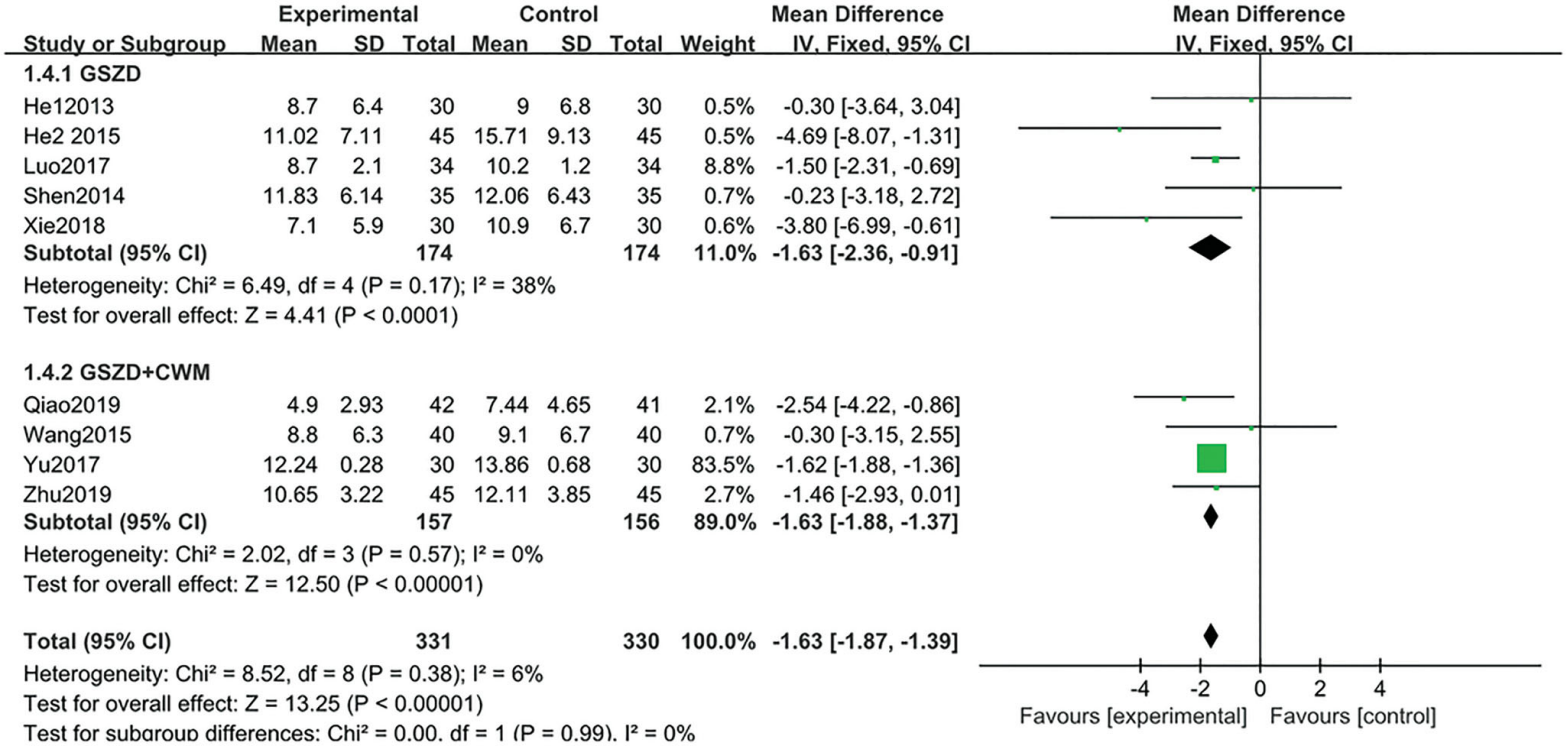
Forest plot of C-reactive protein.

In the aggregate analysis of the nine included trials (He et al. 2013; Shen et al. 2014; He 2015; Wang 2015; Luo 2017; Yu et al. 2017; Xie and Chen 2018; Qiao 2019; Zhu 2019), it was shown that after the treatment, there is a statistically significant decline in the CRP levels in trial groups (GSZD alone and GSZD + CWM), compared to the control group (MD = −1.63, CI = −1.87 to −1.39, *p* = 0.38), and the heterogeneity between studies was not statistically significant (Figure 8). Further sensitivity analysis showed that the combined results of this analysis were highly stable.

**Figure 9.**
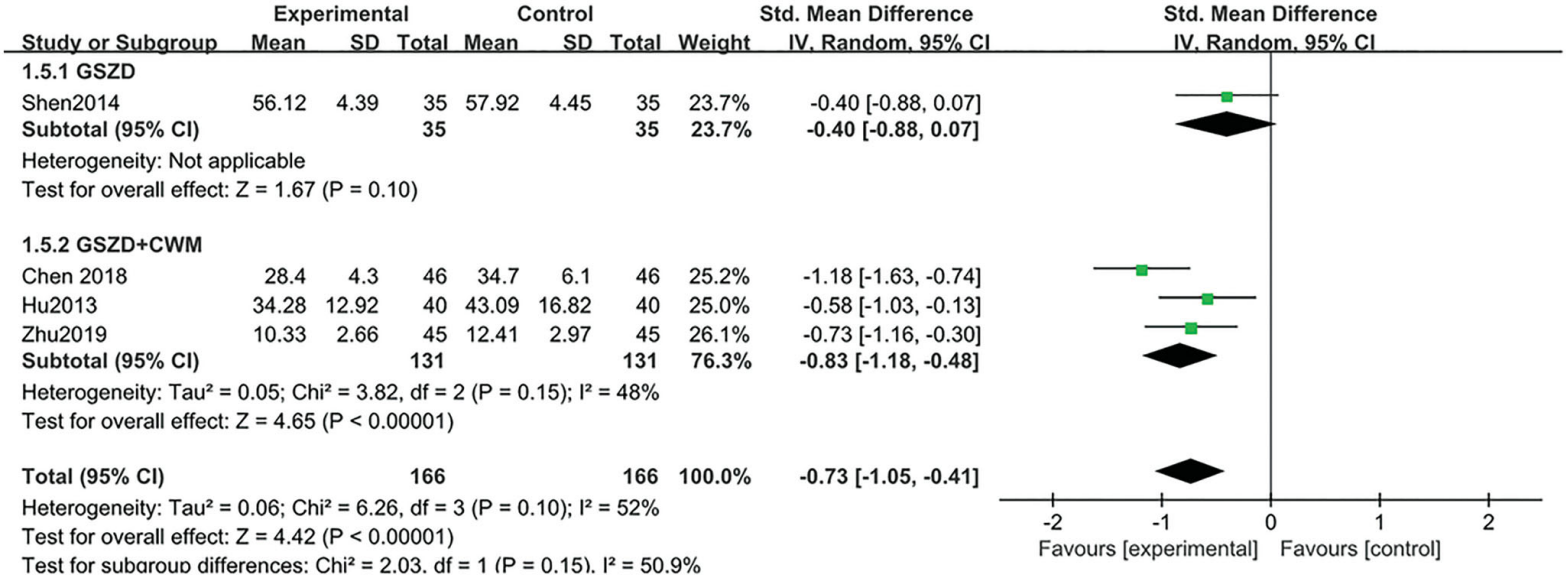
Forest plot of interleukin-6.

Only four studies (Hu and Luo 2013; Shen et al. 2014; Chen 2018; Zhu 2019) evaluated the change of IL-6, and the results indicated that both of GSZD alone or combined with CWM could significantly reduce the IL-6 levels, compared to CWM group (SMD = −0.73, CI = −1.05 to −0.41, *p* = 0.10) (Figure 9). Furthermore, the sensitivity analysis showed that the combined results were stable.

#### Safety evaluation

**Figure 10.**
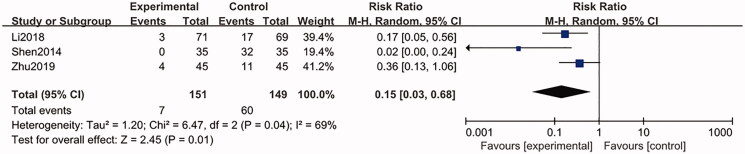
Forest plot of safety evaluation.

Amongst the 13 RCTs, only three studies (Shen et al. 2014; Li et al. 2018; Zhu 2019) recorded the side-effects. One of the three studies (Shen et al. 2014) only reported adverse reactions in the control group, including 29 cases of gastrointestinal reactions, two cases of abnormal blood routine and one case of abnormal function of liver and kidney. For another included trial (Li et al. 2018), adverse reactions occurred in both the trial group and control group. In the control group, five patients had gastrointestinal reactions, eight patients had skin rashes, two patients had liver damage, one patient had thrombocytopenia, one patient had leukopoenia and other bone marrow suppression manifestations, and the reason may be related to the use of allopurinol tablets; for the trial group, three cases of gastrointestinal reactions were observed, which was considered to be involved in the administration of traditional Chinese medicine. In the study of Zhu (2019), eight patients in the control group had gastrointestinal reactions, two patients had abnormal blood routine, one patient had liver injury and four patients in the experimental group had gastrointestinal reactions (Table 2). All the above-mentioned adverse reactions were mild, and all the patients continued to complete the test. The merged results (three trials, 300 participants) showed that GSZD had a lower incidence of side-effect, compared to CWM treatment alone with statistical significance (RR = 0.15, CI = 0.03–0.68, *p* = 0.04) (Figure 10). All the results of statistical analysis were showed in Table 4.

## Discussion

Gout is a kind of metabolic rheumatism which can be complicated with joint destruction and renal impairment. In addition, gout is also often accompanied by hyperlipidaemia, hypertension, diabetes, arteriosclerosis and coronary heart diseases (He et al. 2017). At present, the NSAIDs, colchicine and glucocorticoids, are commonly applied to manage gout. However, long-term use of these drugs may result in several serious side-effects. As an effective ancient oriental medicine, TCMs nowadays show accumulating roles in complementary and alternative therapy for various intractable diseases. GSZD, a classic TCM formula, has been widely used in the clinical treatment of gout for a long time in China, and has been proved to have good clinical efficacy with low side effects. Thus, GSZD seems to be an ideal alternative remedy for gout patients who need lifelong uric acid (UA) lowering treatment. However, it is unfortunate that there was no systematic review for its efficacy in gout treatment so far. By systematic review and meta-analysis of the existing RCTs, we systematically and objectively evaluated the efficacy and safety of GSZD on gout treatment for the first time.

In this meta-analysis, all eligible studies included met the gout diagnostic criteria recognized at home and abroad, and significant clinical indicators for gout treatment were selected as the outcome indicators. In order to reduce the influence of clinical heterogeneity, we also conducted subgroup analysis according to different intervention drugs. Our meta-analysis results showed that the number of patients with gout who achieved clinical efficacy after GSZD treatment was 1.23 times higher than that who received CWM treatment. Meanwhile, GSZD treatment alone could significantly improve UA control and significantly reduce ESR and CRP levels, compared with the CWM treatment alone. In addition, the combined results of studies involving ESR and CRP assessment showed that compared with CWM, GSZD intervention significantly reduced ESR and CRP levels in patients, and the component differences were statistically significant. Additionally, both GSZD group and GSZD + CWM group have better alleviating effects on IL-6 levels than that of CWM group. For safety evaluation, the combined results of the three included trials showed that GSZD treatment has less side-effect than that of the CWM treatment; however due to there is only three trials (Shen et al. 2014; Li et al. 2018; Zhu 2019) could be used for the present safety evaluation, further studies should be devoted to prove the clinical safety of GSZD treatment.

Modern studies have proved that the essential cause of gout is the excessive level of UA in body, which causes excessive deposition of urate crystals in joints and kidney (Miner et al. 2016), and these crystals could further form tophi in the deposition sites, leading to gout. Gout is a recurrent disease with the symptom of severe pain and limited motor functions, which could seriously affect the life quality of gout patients, and gout patients usually require lifelong drop UA treatment. Therefore, how to effectively control the serum level of UA is the predominant strategy for gout treatment. In our meta-analysis, we found that GSZD has a good effect on reduction of UA level in gout patients with fewer adverse reactions, suggesting that GSZD can be used as an alternative remedy for UA management. Furthermore, there is growing evidence that inflammation persists during gout episodes, suggesting that like rheumatoid arthritis, gout is a chronic inflammatory joint disease. The mechanism is that sodium urate crystals induce inflammation by activating neutrophils and inflammatory corpuscles to release similar pro-inflammatory cytokines during acute episodes (Desaulniers et al. 2001). IL-6 is an important member of the interleukin family and plays important roles in the development of inflammation. IL-6 is involved in the immune response of the body by inducing the differentiation of B cells and the production of antibodies, and at the same time inducing the active proliferation and differentiation of T cells, which is the promoter of inflammatory response. In previous pharmacological studies, we found that GSZD has a good effect on improving inflammatory symptoms of rheumatoid arthritis (Zhang et al. 2019). In this meta-analysis, it is reported that GSZD can significantly reduce serum levels of IL-6 in gout patients, suggesting that GSZD can control the inflammatory responses in gout patients. In addition, ERS and CRP are important indicators for clinical evaluation of gout symptoms. At the end of the intervention, GSZD can reverse these indicators and recover to normal level, further demonstrating the clinical effectiveness of gout treatment. Taken together, our present work showed that GSZD is an effective, safe and economic alternative remedy for gout management.

However, the present meta-analysis also has some limitations. First, all the 13 enrolled studies were single-center studies in China, and all of the results were positive, which may have some potential publication deviation risks. Second, small sample size and too short intervention cycle (1–24 weeks, average value was 4.85 weeks) may affect the reliability of conclusions, and the long-term treatment effect of GSZD on gout cannot be evaluated. Third, these RCTs appear to be less rigorous in experimental design, implementation and outcome measurement, which may reduce the study quality. For example, we do not know the random method in some studies, but each study mentioned random distribution. Fourth, the evaluation of the blinding method was not mentioned in each study; this may be due to the particularity and characteristic of individualized treatment based on syndrome differentiation in TCM remedy, and it is difficult to achieve completely blind design in clinical practice. Although the blinding method was not applied, it does not mean that the method of this study is not completely correct (Schulz and Grimes 2006). In addition, the heterogeneity of some of our results cannot be ignored. Reviewing the included studies, we noted that some of the controls used more than one chemical which may be the reason for the heterogeneity. Fifth, the heterogeneity of the results was also affected due to the particularity and characteristic of individualized treatment based on syndrome differentiation in TCM remedy, as well as the different basic conditions of patients, different dosages and different administration time. Fortunately, each study suggested that GSZD had good therapeutic effects against gout. However, multi-centers and large sample clinical trials would be necessary in the future.

## Conclusion

In this systematic review, we comprehensively evaluated the efficacy of GSZD on gout treatment, and found that the efficacy of GSZD in the treatment of gout was better than that of conventional western medicine. However, due to the poor quality and high heterogeneity of the evidence, further studies are needed to confirm this hypothesis.
